# Hemostasis using traction clip closure and self-assembling peptide gel for gastric endoscopic submucosal dissection ulcer

**DOI:** 10.1055/a-2801-5176

**Published:** 2026-03-02

**Authors:** Koichi Soga, Haruka Kato, Hiroki Maeda, Yuki Soma, Ryosaku Shirahashi, Ikuhiro Kobori, Masaya Tamano

**Affiliations:** 1 26263 Department of Gastroenterology, Dokkyo Medical University Saitama Medical Center, Koshigaya, Japan

Delayed bleeding and perforation are major adverse events after gastric endoscopic submucosal dissection (ESD); however, the complete closure of large gastric defects using through-the-scope clips (TTSCs) alone is often difficult 1 2 . Self-assembling peptide (SAP) gel provides rapid hemostasis and may promote mucosal healing 3 . We report the cream puff method, which is a novel prophylactic hemostatic strategy for gastric ESD ulcers.

**Fig. 1:**
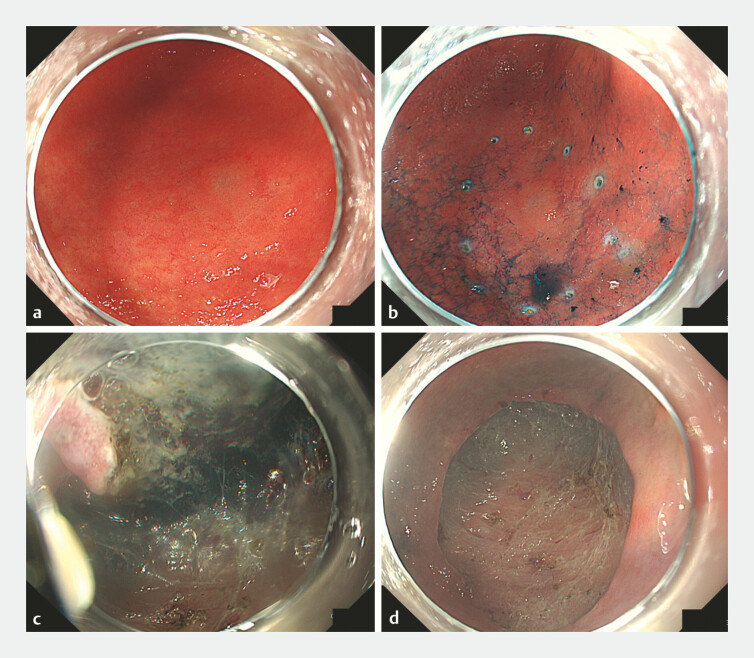
Gastric endoscopic submucosal dissection (ESD) for early gastric cancer. **a** The conventional white light endoscopy image of a 20-mm flat elevated lesion on the greater curvature of the lower gastric body. **b** The indigo carmine chromoendoscopy image clearly delineating the lesion. Dots are placed around the lesion. **c** An intraprocedural view during endoscopic submucosal dissection. **d** The ulcer base after ESD. Exposed vessels were coagulated with hemostatic forceps. Because of the presence of a large gravity-dependent defect, complete closure using conventional clips alone was considered difficult.

A woman in her 70s with multiple comorbidities underwent ESD for a 20-mm flat elevated early gastric cancer. En bloc resection was completed without events. Exposed vessels on the ulcer base were coagulated with hemostatic forceps after ESD; however, a large gravity-dependent defect was considered a high risk factor for delayed complications ( Fig. 1 ).

**Fig. 2:**
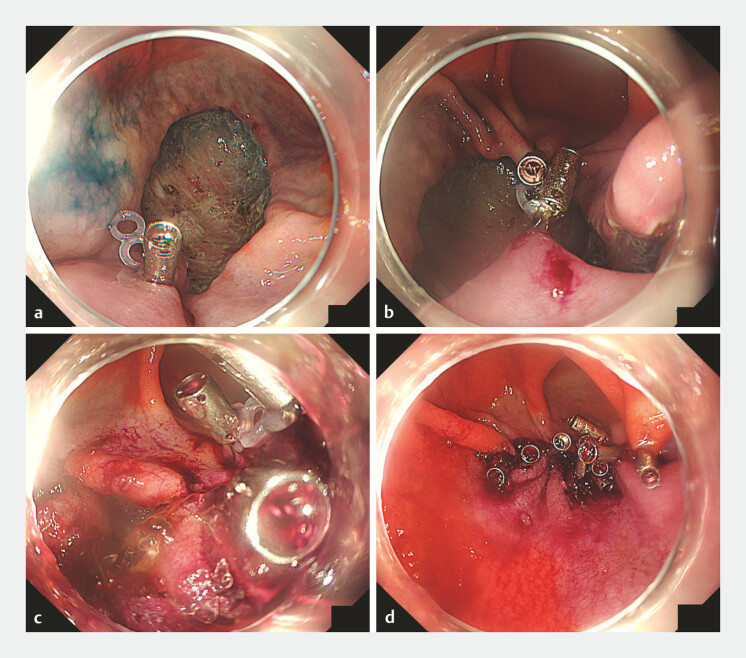
The cream puff method comprising combined traction clip closure and self-assembling peptide (SAP) gel after gastric endoscopic submucosal dissection (ESD). **a** A traction clip with silicone bands is applied to healthy mucosa at the oral ulcer edge. **b** A second clip is placed on the anal edge while pulling the silicone band, thereby approximating the oral and anal edges. Additional clips are placed along the approximated margins, resulting in the closure of approximately two-thirds of the defect and formation of a small residual pocket. **c** SAP gel (Purestat; 3-D Matrix Ltd, Tokyo, Japan) is injected into the pocket through a catheter. The ulcer base and underside of the approximated mucosa are filled with gel in a manner similar to that used to fill a cream puff. **d** The final appearance after additional clips were applied to close the remaining opening, complete mucosal approximation, and seal the gel-filled pocket.

A TTSC equipped with silicone traction bands was placed on healthy mucosa at the oral edge of the ulcer. A second TTSC was placed on the anal edge while pulling the band, thereby approximating both edges and decreasing the exposed ulcer bed area. Additional TTSCs were applied between the approximated edges. Approximately two-thirds of the ulcer circumference was closed and a narrow residual pocket was created. Subsequently, 1 mL of the SAP gel (Purestat; 3-D Matrix Ltd, Tokyo, Japan) was injected into the pocket through a catheter to fill the ulcer base and underside of the reapproximated mucosa. This technique is similar to that used to inject cream into a cream puff. Additional TTSCs were placed to close the remaining opening and seal the gel-filled pocket ( Fig. 2 and Video 1 ). Delayed bleeding, perforation, and infection did not occur during the postoperative course.

An endoscopic sequence demonstrating the cream puff method comprising traction clip-assisted closure and self-assembling peptide (SAP) gel injection for prophylactic hemostasis after gastric endoscopic submucosal dissection.Video 1

The cream puff method combines stable mechanical closure with prolonged SAP gel retention to effectively reduce the functional dead space, delay complications, and support wound healing. Therefore, it may be a feasible option for the prophylactic hemostasis of high-risk gastric ESD ulcers.

Endoscopy_UCTN_Code_TTT_1AO_2AD
